# 中国肺癌低剂量CT筛查指南（2023年版）

**DOI:** 10.3779/j.issn.1009-3419.2023.102.10

**Published:** 2023-01-20

**Authors:** Chinese Expert Group on Early Diagnosis and Treatment of Lung Cancer, China Lung Oncology Group

**Keywords:** 肺肿瘤, 筛查, 低剂量计算机断层扫描, 指南, Lung neoplasms, Screening, Low-dose computed tomography, Guideline

## Abstract

肺癌是导致中国癌症死亡的首要原因。近年来低剂量计算机断层扫描（low-dose computed tomography, LDCT）筛查的效果进一步被证实，并且在高危人群选择、筛查间隔及结节管理的研究方面取得了显著进展。本研究的目的是对2018年中国肺癌LDCT筛查指南进行修订。由国家卫健委任命的中国肺癌早诊早治专家组专家及中国西部肺癌研究协作中心部分专家，共同参与了2023版中国肺癌筛查指南的修订工作。专家们根据近年来国内外LDCT肺癌筛查进展，结合我国肺癌流行病学特征，共同修订了本次肺癌筛查指南。本指南对以下方面进行了修订：（1）高危人群定义中考虑了除吸烟外其他肺癌危险因素；（2）对LDCT扫描参数进行了修改和补充；（3）扩大了部分筛查阴性个体的筛查间隔；（4）将部分阳性结节的随访时间由3个月调整为6个月；（5）强调了多学科诊疗（multi-disciplinary treatment, MDT）在阳性结节管理、肺癌诊断和治疗中的作用。本次修订将使得LDCT筛查指南更适应我国国情，并使筛查、干预与治疗路径更为明确。未来应进一步基于新兴技术，包括生物标志物及人工智能研究，优化肺癌LDCT筛查方法及技术。

## Abstract (en)

Lung cancer is the leading cause of cancer-related death in China. The effectiveness of low-dose computed tomography (LDCT) screening has been further validated in recent years, and significant progress has been made in research on identifying high-risk individuals, personalizing screening interval, and management of screen-detected findings. The aim of this study is to revise China national lung cancer screening guideline with LDCT (2018 version). The China Lung Cancer Early Detection and Treatment Expert Group (CLCEDTEG) designated by the China's National Health Commission, and China Lung Oncolgy Group experts, jointly participated in the revision of Chinese lung cancer screening guideline (2023 version). This revision is based on the recent advances in LDCT lung cancer screening at home and abroad, and the epidemiology of lung cancer in China. The following aspects of the guideline were revised: (1) lung cancer risk factors besides smoking were considered for the identification of high risk population; (2) LDCT scan parameters were further classified; (3) longer screening interval is recommended for individuals who had negative LDCT screening results for two consecutive rounds; (4) the follow-up interval for positive nodules was extended from 3 months to 6 months; (5) the role of multi-disciplinary treatment (MDT) in the management of positive nodules, diagnosis and treatment of lung cancer were emphasized. This revision clarifies the screening, intervention and treatment pathways, making the LDCT screening guideline more appropriate for China. Future researches based on emerging technologies, including biomarkers and artificial intelligence, are needed to optimize LDCT screening in China in the future.

## 1 前言

肺癌仍是我国癌症发病率和死亡率最高的恶性肿瘤^[1]^。据估计，2016年我国新发肺癌为82.8万例，发病率为59.89/10^5^，死亡例数65.7万，死亡率为47.51/10^5^。肺癌发病率和死亡率非常接近，说明其预后较差。从2003年-2005年至2012年-2015年，我国肺癌的5年生存率仅从16.1%提高到19.7%^[2]^，主要原因仍是未能做到早期诊断和早期治疗。肺癌的生存与分期密切相关，分期越早，预后越好，I期肺癌患者的5年生存率可达85.5%-90.2%，但我国肺癌患者在诊断时为I期的比例低于20%^[3,4]^。因此，肺癌筛查和早期诊断对于改善患者预后、降低肺癌死亡率具有重要意义。

2011年，美国国家肺癌筛查试验（National Lung Screening Trial, NLST）首次证明低剂量计算机断层扫描（low-dose computed tomography, LDCT）筛查可显著降低肺癌死亡率^[5]^。近年来，欧洲的荷兰-比利时肺癌筛查试验（Nederlands-Leuvens Longkanker Screenings Onderzoek, NELSON）、德国和意大利的肺癌筛查试验也进一步证实了LDCT肺癌筛查的效果^[6⇓-8]^。目前，我国LDCT肺癌筛查的开展也日益广泛，自2010年起先后启动了农村和城市肺癌筛查项目，各省市也开展了一些LDCT肺癌筛查的公共卫生服务或研究^[9⇓-11]^。2018年，我们基于NLST的研究结果及我国肺癌筛查技术方案制定了《中国肺癌低剂量螺旋CT筛查指南（2018年版）》^[12]^。此后，国内外为降低LDCT筛查的不确定性和潜在危害，在高危人群选择、结节管理、筛查间隔等环节开展了大量的研究，并对人工智能、深度学习等多种新技术在肺癌筛查中的应用进行了探索^[13]^。本研究将根据近年来国内外LDCT筛查进展，对2018年版指南进行修订。

## 2 背景

近年来，多项随机对照试验报道了肺癌LDCT筛查效果评价的结果。2020年，欧洲NELSON试验^[6]^经过10年的随访发现，与对照组相比，LDCT筛查使男性高危人群的肺癌死亡率显著下降（24%），而女性肺癌死亡率降低幅度虽然更大（33%），但无显著意义。意大利、英国、德国的4项随机对照试验^[7,8,14,15]^的结果也显示了筛查组肺癌死亡率的降低，但由于样本量较小，均未达显著差异。Meta分析和系统评价^[15,16]^的结果表明，与对照组比较，LDCT筛查可使肺癌死亡率显著降低16%-21%。此外，我国一项多中心前瞻性队列研究^[17]^基于逆概率加权比较了筛查人群和未筛查人群的肺癌死亡率，认为单次LDCT筛查即可显著降低31%的肺癌死亡率。但此研究并非随机对照研究，仍存在争议^[18]^。

2018年，美国癌症协会对2013年筛查指南进行了更新，建议55岁-74岁、吸烟史≥30包年的吸烟者或戒烟个体（戒烟时间<15年）进行筛查。2021年，美国预防服务工作组开展了一项对LDCT肺癌筛查准确性、获益及危害的系统评价^[19]^，并基于此系统评价对其肺癌筛查指南进行了更新。此次更新扩大了肺癌筛查的高危人群范围，将筛查的年龄从55岁降至50岁，并将吸烟史的标准从30包年降至20包年，建议50岁-80岁、吸烟史≥20包年的目前吸烟者或戒烟不足15年者接受年度性的LDCT筛查^[20]^。2022年，美国国立综合癌症网络（National Comprehensive Cancer Network, NCCN）将肺癌筛查指南更新为2023.V1版本^[21]^。英国、德国等其他一些国家也于近些年发布了关于肺癌筛查的指南或建议。我国国家癌症中心于2021年制定了我国的肺癌筛查标准和指南^[22,23]^。

## 3 参与本指南修订的人员结构及修订方法

本指南由中国肺癌早诊早治专家组和中国西部肺癌研究协作中心的部分肺癌专家基于2011年肺癌早诊早治技术方案、2015年和2018年肺癌筛查指南进行修订^[12,24]^。肺癌早诊早治专家组成员（由国家卫健委指定）和来自中国西部肺癌研究协作中心的专家，专业背景涵盖胸外科、肿瘤内科、影像学、病理学、流行病学等学科。

与2018年版指南类似，本指南基于2018年以后新报道的肺癌筛查随机对照试验结果、LDCT筛查系统评价、我国LDCT城市和农村肺癌筛查实践、2018年后新的国外筛查指南或指南的更新。基于对LDCT筛查获益、危害、高危人群、筛查间隔及结节管理的评估，对本次指南进行了修订。

## 4 知情同意与共同决策

鉴于LDCT筛查存在辐射危险和假阳性等潜在危害，建议高危个体在参加筛查前与医疗专业人员就LDCT筛查的益处和危害进行权衡和讨论，共同决策。

## 5 高危人群的选择及筛查间隔

目前的肺癌筛查研究和指南中，仍主要是参照美国肺癌筛查试验根据年龄和吸烟选择高危人群，并进行相应的调整。但目前对筛查开始年龄、停止年龄以及吸烟的暴露量和戒烟时间的建议并不一致。筛查的开始年龄和停止年龄，分别为50岁-55岁和70岁-80岁。吸烟量介于15包年-30包年，戒烟最短时间为5年。目前，尚无证据支持开始和停止筛查的具体年龄。在NLST试验中，高危人群的纳入标准为：年龄为55岁-74岁，至少有30包年吸烟史，且如果戒烟，则戒烟时间<15年。在欧洲NELSON试验中，筛查个体年龄在50岁-74岁，吸烟史超过25年且每天超过15支，或吸烟史超过30年且每天超过10支，如果曾经戒烟，戒烟时间在10年内。2021年，美国预防服务工作组对肺癌筛查指南进行了修改，将高危人群筛查开始年龄从55岁降至50岁，将吸烟暴露从30包年降至20包年，从而可使更多的吸烟暴露较低但仍具有肺癌风险的人受益于筛查，包括更多的女性和少数种族或民族。

我国肺癌发病的危险因素较国外更为复杂：（1）我国和西方国家人群对吸烟的易感性不同，与不吸烟者相比，我国吸烟者发生肺癌的相对危险度显著低于西方人群^[25]^；（2）与其他国家相比，严重的空气污染、生物燃料的使用等其他危险因素对我国肺癌的影响更大^[9]^。因此，在本次修订中，我们将除吸烟外的其他危险因素也考虑在内。如果具备下列条件之一，则建议参加肺癌筛查：（1）年龄介于50岁-80岁；（2）具有下列条件之一：①吸烟史：吸烟≥20包年（每天吸烟包数×吸烟年数）或被动吸烟≥20年，若现在已戒烟，戒烟时间不超过5年；②有长期职业致癌物暴露史：长期接触氡、砷、铍、铬及其化合物，石棉，氯甲醚，二氧化硅，以及焦炉逸散物和煤烟等肺癌致癌物；③一级、二级亲属患肺癌，同时吸烟≥15包年或者被动吸烟≥15年；④如果某些高发地区有其他重要的肺癌危险因素也可作为筛选高危人群的条件。LDCT筛查的禁忌证与2018年版筛查指南相比无变化。

## 6 LDCT扫描参数

本指南LDCT扫描参数建议如下。

### 6.1 采用CT容积扫描技术

管电压采用110 KVp，管电流40 mAs，依据受试者体重，根据不同身体质量指数（body mass index, BMI）做一定范围内的调整。对于BMI≤30 kg/m^2^受检者，建议扫描条件为管电压100 kVp-120 kVp，管电流≤40 mAs，总剂量≤0.2 mSv；对于体型较大的受检者（BMI>30 kg/m^2^），建议扫描条件为管电压120 kVp，管电流≤60 mAs，总剂量≤0.5 mSv。

### 6.2 扫描范围

从肺尖到肋膈角（包括全部肺），患者吸气末一次屏气完成扫描。

### 6.3 扫描后原始数据分析

行薄层重建，重建层厚为0.625 mm-1.250 mm。为方便进行计算机辅助检测（computer aided diagnosis, CAD）及容积分析，建议层间有20%-30%重叠。

### 6.4 薄层重建算法

建议采用软组织密度或肺算法，不建议采用高分辨率骨算法，后者对软件容积分析重复性影响较大。

### 6.5 肺结节的检测建议

将薄层图像行三维重建，采用最大密度投影（maximal intensity projection, MIP）重建，有助于结节的检出及结节形态的观察。推荐应用CAD软件结合人工阅片，提高结节检出率。

### 6.6 图像后处理方法

对可疑肺癌的微小磨玻璃结节采用多平面重组（multiplanar reformation, MPR）、MIP、曲面图像重组（curved planar reformation, CPR）进行图像后处理，这能更有利于观察结节内部血管结构、周围边缘及移动血管的显示，以明确是否符合小肺癌的影像诊断。

## 7 LDCT肺癌筛查间隔

目前对于1年是否为LDCT筛查的最佳间隔仍存在争议。包括NLST和欧洲NELSON试验的研究^[26,27]^表明，筛查阴性个体后期的肺癌发生风险往往较低。因此，本次修订中，建议连续2年筛查阴性的人群停止筛查2年，筛查阳性的人群需每年筛查。

## 8 结节管理

LDCT筛查检出的结节中，大部分为良性结节。因此如何定义阳性结节、阳性结节如何进一步鉴别分流对于降低LDCT过高的假阳性至关重要。然而，如何确定结节的最佳管理方案来区分良恶性质目前尚无定论。NLST和NELSON试验^[5,6]^均证明LDCT筛查可降低肺癌死亡率，但两项试验中阳性结节的定义和管理差别较大。NLST中，直径4 mm及以上的结节需接受进一步检查，而NELSON试验则根据体积倍增时间对结节进行管理。本指南中，结节的管理方案采用了中国农村肺癌早诊早治项目的技术方案。

### 8.1 阳性结节的定义

LDCT筛查发现的结节可分为两大类：（1）肯定良性结节或钙化性结节；（2）不确定结节或非钙化性结节，此类结节根据结节性质及大小确定随访原则，并根据随访中结节的生长特性确定是否进行临床干预。

基线筛查：若实性结节或部分实性结节直径≥5 mm，或非实性结节直径≥8 mm，或发现气管或/及支气管可疑病变，或LDCT诊断为肺癌的肺部单发、多发结节或肺癌包块，应当进入临床治疗程序，则定义为阳性。

年度筛查：发现新的非钙化性结节或气道病变，或发现原有的结节增大或实性成分增加，则定义为阳性。

### 8.2 结节管理和进一步诊断

#### 8.2.1 基线筛查检出的结节（图1）

**图1:**
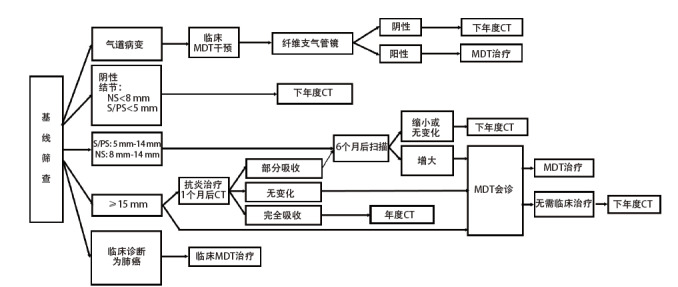
基线筛查流程及结节管理。CT：计算机断层扫描；MDT：多学科诊疗；NS：非实性结节；S：实性结节；PS：部分实性结节。

CT检查阴性者，直径<5 mm的实性结节或部分实性结节，以及直径<8 mm的非实性结节：12个月后按计划进入下一年度复查。

直径5 mm-14 mm的实性结节或部分实性结节以及直径8 mm-14 mm非实性结节：筛查后6个月进行复查。如果结节增大，由临床多学科诊疗（multi-disciplinary treatment, MDT）团队会诊，决定是否进入临床MDT治疗；如结节无变化或缩小，进入下一年度复查。对于直径≥15 mm结节，有两种方案：（1）由临床MDT团队会诊，决定是否进入临床MDT治疗；（2）抗炎治疗2周-3周，休息1个月后复查。如果病灶完全吸收，进入下一年度复查；如果结节无变化，由临床MDT会诊，决定是否进入临床MDT治疗；如果结节部分吸收，6个月后进行CT复查，结节增大者，由临床MDT会诊，决定是否进入临床MDT治疗，结节缩小或无变化者，进入下一年度复查。

LDCT诊断为肺癌的肺部单发、多发结节或肺癌包块，应当进入临床MDT治疗程序。LDCT筛查如发现气管和/或支气管可疑病变，应进行临床MDT治疗，并进行纤维支气管镜检查，并在必要时进一步随诊。

#### 8.2.2 年度筛查结节管理（图2）

**图2:**
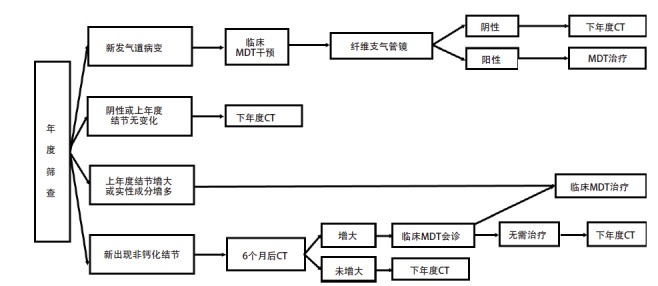
年度筛查流程及结节管理

对于年度CT复查发现的新的非钙化性结节，6个月后复查，如果复查结节增大，由临床MDT团队决定是否进入MDT治疗；如果未增大，进入下一年度复查。

年度复查发现原有的肺部结节明显增大或实性成分明显增多时，应进入全程管理的临床MDT治疗程序。

年度筛查中发现的气管和/或支气管可疑病变，处理同基线筛查。

### 8.3 临床干预

包括以下几种情况：（1）LDCT检查发现气道病变者，应该施行纤维支气管镜检查。纤维支气管镜检查阳性且适合外科手术治疗者，应当施行以外科手术为主的MDT综合治疗。纤维支气管镜检查阴性者则进入下一年度复查。（2）LDCT诊断为肺癌或高度疑似肺癌者：①LDCT筛查高度怀疑为肺癌的肺部阳性结节者，应当由高年资的胸外科、肿瘤内科、呼吸科和影像医学科医师进行MDT会诊，决定是否需要进行临床治疗以及采取什么方法进行治疗。对于适合外科手术治疗者，首选外科治疗。②LDCT诊断为肺癌的肺部单发、多发结节或肺癌包块，应当进入临床治疗程序，经临床检查适合外科手术治疗者，施行以外科手术为主的MDT治疗。（3）LDCT诊断为肺癌或高度怀疑为肺癌的肺部单发、多发结节或肺部包块，由于肿瘤原因、患者心肺功能异常不能耐受外科手术治疗，或者患者本人不愿意接受外科手术治疗者，为明确病变性质进行纤维支气管镜细胞学刷检、活检，或者跨支气管壁纵隔淋巴结穿刺活检等病理学细胞学检查；对于通过纤维支气管镜检查不能获得病理学或者细胞学诊断者，可以施行经皮肺穿刺活检标本送病理检查。上述方法获得的肿瘤组织标本，除了做病理学或者细胞学检查外，应当做下一代测序（next-generation sequencing, NGS）检查，以便确定患者是否适合分子靶向治疗。通过上述方法获得肺癌病理学、细胞学检查明确肺癌诊断及驱动基因检测结果后，应当组织MDT讨论，给予肺癌治疗全程管理的“个体化”MDT治疗。

## 9 筛查与戒烟结合

戒烟是降低肺癌死亡率的最有效的干预措施。作为肺癌的二级预防措施，LDCT肺癌筛查并不能替代戒烟，而是需要将戒烟与肺癌筛查紧密结合，在筛查实践中大力推行戒烟措施。

## 10 讨论

与2018年版本相比，本版本根据近年来国内外LDCT肺癌筛查进展，结合我国肺癌流行病学特征，对以下方面进行了修订：（1）高危人群定义中考虑了除吸烟外的其他肺癌危险因素；（2）对LDCT扫描参数进行了修改和补充；（3）扩大了部分筛查阴性个体的筛查间隔；（4）将部分阳性结节的随访时间由3个月调整为6个月；（5）强调了MDT在阳性结节管理、肺癌诊断和治疗中的作用。本次修订将使得LDCT筛查指南更适应我国国情，并使筛查、干预与治疗路径更为明确。

本次修订中，我们将吸烟外的肺癌危险因素也考虑在内。一方面，尽管吸烟是肺癌最重要的危险因素，但吸烟者中只有5%-10%有可能罹患肺癌，仅根据年龄和吸烟选择高危个体准确性较低；另一方面，与西方国家相比，我国肺癌发病的危险因素更为复杂，超过40%的肺癌发生于非吸烟者中。因此，在定义我国肺癌高危人群标准时，有必要将这些吸烟以外的因素也考虑在内^[28⇓-30]^。近年来，多项国外研究基于年龄、性别、种族、教育水平、BMI、家族史、吸烟史等因素建立了多个肺癌风险预测模型，包括Bach模型、Spitz模型、LLP模型、PanCan模型、PLCO模型等，并发现这些风险预测模型可更为精准地选择适合LDCT肺癌筛查的高危人群^[31]^。此外，几项研究^[32⇓-34]^在不同人群中对部分上述风险预测模型的效能进行了比较，但结果并不一致。近期美国预防服务工作组的LDCT肺癌筛查系统评价中也对肺癌风险预测模型在肺癌筛查中的效能进行了评估，不同风险模型间也存在差异，因此，在2021年美国预防服务工作组肺癌筛查指南^[19,20]^中，未建议基于风险预测模型进行高危人群分层。

由于国内外肺癌流行病学及临床特征的差异，基于中国人群样本建立适合于中国人群的肺癌风险预测模型，尤其是纳入非吸烟高危个体，精准筛选最适合于LDCT筛查的高危人群是我国肺癌筛查目前亟需解决的问题之一。我国一项研究^[35]^基于国家肺癌筛查项目超55万人的多中心队列，建立了包含非吸烟者和吸烟者的肺癌3年风险预测模型（中国NCC-LCm2021模型）。此模型在另外两项前瞻性队列中进行了外部独立验证，在非吸烟者和吸烟者中的受试者工作特征曲线下面积分别为0.698、0.673和0.728、0.752。研究者同时将此模型与国外多个模型在验证队列中进行了比较，发现其准确性更高，且具有良好的校准度。NCC-LCm2021是国内首个基于国内大规模队列建立并进行外部独立验证的肺癌风险预测模型，并提出了肺癌风险的阈值来定义高危个体，但此研究随访时间较短，且未来也需要更多的验证。未来我们也将根据后期的研究结果来对高危人群的选择进行修订。

在筛查指南和大部分筛查实践中，肺癌筛查间隔均为1年。但目前对于1年是否为LDCT筛查的最佳间隔仍存在争议^[36⇓-38]^。NLST和NELSON试验、我国农村肺癌筛查的多项研究^[26,27,39]^表明，筛查阴性个体后期的肺癌发生风险往往较低。一项基于NLST试验的回顾性分析^[26]^发现，与整个LDCT筛查组相比，LDCT组基线筛查阴性个体的肺癌发病率、死亡率和肺癌检出率均显著降低。类似地，我国农村肺癌筛查项目个旧项目点基线筛查阴性个体的肺癌发病率也远远低于整个筛查人群和美国NLST标准^[39]^。研究^[40,41]^表明，将个体特征与既往筛查结果结合，有助于更精准地预测LDCT筛查阴性个体的肺癌发病风险，从而合理扩大低危个体的筛查间隔。鉴于频繁筛查所带来的潜在危害，延长这部分个体的筛查间隔有可能减少筛查的潜在危害，提高筛查效率。在本次修订中，我们建议连续2年筛查阴性个体可停止筛查2年。

本次修订对LDCT扫描参数进行了修改和补充，并将部分阳性结节的随访时间由3个月调整为6个月，目的也是减少LDCT筛查的潜在危害，包括辐射相关的风险及假阳性相关的过度检查、过度治疗^[42]^。同时，考虑到LDCT筛查的潜在危害，本次修订也继续强调了个体的知情同意权和与医疗专业人员的共同决策过程。

戒烟和筛查相结合，可以使筛查获益更大^[43]^。在NLST试验中，戒烟15年联合LDCT筛查可使肺癌死亡率下降38%^[44]^。多项研究^[45⇓-47]^探讨了LDCT肺癌筛查对戒烟的效果，但尚无统一结论。一项有关我国农村肺癌筛查项目的研究^[48]^发现LDCT筛查同时具有“戒烟教育”和“吸烟许可”的作用，但总体上有助于筛查人群吸烟率的降低。将来应该开展更多研究探讨不同筛查结果对戒烟的影响，以及通过哪些策略强化LDCT筛查与戒烟的联合效果。

分子标志物、影像组学、人工智能等新兴技术的发展，有助于从高危人群选择、筛查间隔及阳性结节鉴别分流等方面优化肺癌筛查策略。我国一项研究^[49]^在前期中国人群肺癌全基因组关联研究的基础上，基于19个独立易感位点构建了中国人群肺癌多基因遗传风险评分PRS-19，可有效用于肺癌高危人群的选择。随着大数据的应用、理论算法的革新以及计算能力的显著提高，人工智能也逐渐用于肺癌的风险评估及结节的检测与诊断。国外一项研究^[50]^基于NLST试验的CT图像数据，开发并验证了名为“Sybil”的深度学习模型，此模型通过一次LDCT扫描预测个人未来的肺癌风险，从而有助于个体化的肺癌筛查。另一方面，分子标志物和LDCT筛查的联合应用、CT图像的影像组学和人工智能分析也有助于降低影像学筛查过高的假阳性结果^[51,52]^。但目前研究中报道的大量肺癌早期标志物及人工智能模型尚处于探索阶段，其作用尚需进一步验证。

## 11 结语

肺癌仍是我国第一大癌症，在NLST之后，近年来NELSON试验和系统评价进一步证实了LDCT肺癌筛查降低肺癌死亡率的效果。此外，国内外在肺癌高危人群选择、筛查间隔、结节管理等方面取得了一系列进展，从而为LDCT肺癌筛查方案的优化、减少其潜在危害提供了科学依据。基于上述研究进展及国内外肺癌筛查指南，希望本次筛查指南的修订能够有助于提高我国LDCT筛查实践获益，减少其潜在危害。
